# Supplementary material for: Characterization of aminoglycoside resistance genes in multidrug-resistant Klebsiella pneumoniae collected from tertiary hospitals during the COVID-19 pandemic
Source: PLoS One. 2023 Jul 28;18(7):e0289359. doi: 10.1371/journal.pone.0289359 (PMC10381092; doi:10.1371/journal.pone.0289359)
Supplement: Attachment — Submitted filename: author response to reviewrs.docx [file pone.0289359.s001.docx]

**Comments:**

| **journal requirements:** | **response:** |
| --- | --- |
| Please ensure that your manuscript meets PLOS ONE's style requirements. | DONE  Formatted by AJE  www.aje.com (see certificate) |
| We suggest copyedit your manuscript for language usage grammar (AJE). | DONE  copyedit by AJE  www.aje.com (see certificate) |
| previous publication(s), which needs to be addressed: | done (at end of discussion) |
| All PLOS journals require that the minimal data set be made fully available | will be provided upon request done |
| 5. PLOS ONE now blot or gel results reported in a submission’s figures or Supporting Information files. | done |
| Additional Editor Comments:  1. In the discussion section, the authors should kindly provide the interpretation of their study results while comparing these to other published works and do not repeat the results in the discussion section. | corrected |
| 2. The authors need to highlight the limitations of their study.  3. There are two concluding sections in this manuscript. The authors need to merge these into one conclusion section, please. | limitation was highlighted and conclusions were merged |
| **Reviewers' comments:** | |
| 1. Is the manuscript technically sound, and do the data support the conclusions? Reviewer #1: Partly  Reviewer #2: Yes  Reviewer #3: Partly | OK |
| 2. Has the statistical analysis been performed appropriately and rigorously?  Reviewer #1: No  Reviewer #2: Yes  Reviewer #3: Yes | OK |
| 3. Have the authors made all data underlying the findings in their manuscript fully available? Reviewer #1: Yes  Reviewer #2: Yes  Reviewer #3: No | OK |
| 4. Is the manuscript presented in an intelligible fashion and written in standard English? Reviewer #1: No  Reviewer #2: No  Reviewer #3: No | DONE  English grammar was edited by AJE  www.aje.com (see certificate) |
| **Reviewer #1:  Here are some comments to help improve the quality of the manuscript.** |  |
| 1. Line 2, page 8; please use coronavirus disease (COVID-19) pandemic instead of coronavirus disease -19 (COVID-19) | Done |
| 2. Line 3, page 8; the aim of the study does not correspond to the title | Edited |
| 3. Line 6, page 8; please remove “overall” | Removed |
| 4. Line 7, page 8; please correct “Of” to “of” | Corrected |
| 5. Line 12, page 7; please use the word “respectively” and not “each” | Done |
| 6. Line 12, page 8; please change “less frequent” to “least frequent” | Done |
| 7. Line 11, page 8; is there any reason that sentence is italicized? If none, please change to regular letters | Changed |
| 8. Line 11, page 8; please maintain consistency in the use of denominators throughout the manuscript it would help in clarifications | Done |
| 9. Line 13, page 8; please review this statement. As stated in your findings, is the increase as a result of increased reporting followed a none or decreased reporting era or an actual increase in numbers during this period? | An actual increase in numbers during this period |
| 10. Line 14, page 8; please review the use of the word “emerging” | Done |
| 11. Line 15, page 8; please change statement to “for continuous implementation of effective infection prevention control (IPC) measures…..” | Changed |
| 12. Line 17, page 8; please revise and or review the inclusion of COVID-19 pandemic as a keyword. The study does not show strong attribution to coronavirus disease (SARS-COV-2) unless if the 220 samples were from ONLY COVID-19 positive patients or if the use was just to describe an era? This was not stated in the description of your samples | Reviewed and preferred to be included |
| 13. Line 25, page 8; please remove 2019 and leave as coronavirus disease (COVID-19) | Done |
| 14. Line 28, page 8; please include SARS-COV-2 in bracket after the full name | Done |
| 15. Line 32, page 8; please change “Europe countries” to “Europe” | Done |
| 16. Line 44, page 9; please correct “others” to “other” | Done |
| 17. Line 45, page 9; please remove “also” so it reads “Other mechanisms” | Done |
| 18. Line 56, page 9; please remove “aimed to identify” it is a tautology | Done |
| 19. Lines 61-64, page 9; there is no proper description as to the nature of the isolates for example were they from only COVID-19 positive cases, were they outpatients or inpatients or from ICU patients hence was it a healthcare associated infection or community acquired these would shed more light to the correlation | clarified |
| 20. Line 64, page 9; please what disease were you referring to in this sentence for clarity | clarified |
| 21. Line 68, page 10; please use acceptable short forms for units. 30 min should be 30 mins or 30 minutes | Done |
| 22. Line 70, page 10; please use acceptable short forms for units 3 h should be 3 hrs. or 3 hours | Done |
| 23. Lines 81-85, page 10; please rewrite and give clarity to the statement | Done |
| 24. Line 93, page 10; please use acceptable short forms for units. 10 s should be 10 secs or 10 seconds. please be uniform throughout your writing this includes line 99 and any others | Done |
| 25. Line 93, page 10; please correct “the isolates then were” to “the isolates were then” | Corrected |
| 26. Line 108, page 11; 220 should be in figures not words, write numbers in figures not words | Done |
| 27. Line 115, page 11; the use of the phrase “elevated resistance rates” should be reviewed since there is no comparism (with a previous result or another study). The phrase “high resistance rate” is more appropriate in this case. | Done |
| 28. Line 117, page 11; please use the word “total” in place of “complete” | Done |
| 29. Line 121-125, page 11; the name K. pneumoniae should be in italics | Done |
| 30. Line 126, page 11; please replace the word “less” with “least” | Done |
| 31. The discussion section should be rewritten please, with clarity on aim of the study and the title and the importance of COVID-19 in the study. As stated in the discussion, is the increase as a result of increased reporting (in this case due to active case search) or an actual increase in numbers of cases with time? | done |
| 32. It may also help to review the references and use more recent ones with SARS-COV-2 involved directly to bring out a strong attribution. Of the 33 references used, only 8 were from the COVID pandemic era (2020-2021) and only 4 of the 8 had COVID-19 positive cases this is important based on your title. | Four references were added (33,34, 35, 37) |
| The results should not be repeated but interpreted and compared to other studies to further strengthen your aim. | improved |
| Some sentences are incomplete or may be contradictory e.g., “whereas MDR K. pneumoniae may be associated with both COVID-19 ICU and non-COVID-19 ICU patients [12] [13] [14]. | Improved |
| The frequent use of antibiotics and extended ICU stays were identified as common risk factors for outbreak situations. Moreover, the excessive use and misuse of antibiotics for viral respiratory infection may be the reason for elevated resistance rates” please shed more light | Done (last of introduction) |
| 33. Page 16, Conclusion; please correct the statement “there are need in continuous implementation of effective control measures” to “Therefore implementation of effective infection prevention control measures should be continuous in order to monitor the occurrence of MDR pathogens in such situations”, or use “there is need.’. Is this statement suggestive that IPC measures be restricted to COVID-19? Kindly provide some recommendations based on your findings also the data provided should support the title of the study | done |
| 34. If this research is to be published, the title should be reviewed; the attribution to COVID-19 is weak. Though samples were collected during the COVID-19 pandemic nothing strong suggesting resistance occurred as a result of coronavirus infection. | The authors prefer TO keep the title as it is, as the purpose of the study was to evaluate the prevalence of aminoglycoside resistance gene during era of COVID-19 pandemic. |
| 35. The work could be presented without the unnecessary attribution to COVID-19 except the isolates were strictly from ONLY COVID-19 positive cases which wasn’t stated. | The purpose of the study was to evaluate the prevalence of aminoglycoside resistance gene during era of COVID-19 pandemic, targeting any patient infected with Klebsiella pneumoniae with COVID-19 infection or without infection, we concentrate on period rather than the case |
| **Reviewer #2:  Areas for improvement:** |  |
| The authors should read the article carefully to correct all grammatical errors and typos e.g. Line 3 under PCR Analysis has "descried" instead of described, The last sentence under Introduction has “ identify aimed to identify the”… and many others. | done |
| There appears to be a mix-up of tables 1 and 2 in the description under the results section. | Corrected |
| The authors should check the frequencies and percentages of one or more aminoglycoside genes and the single gene detected in the MDR K pneumonia – 35/42 and 7/42 as opposed to 35/51 and 7/51. | DONE  English grammar was edited by AJE  www.aje.com (see certificate) |
| There were two concluding sections. | merged |
| No limitations of the study were identified for example PCR has its limitations - specificity, sensitivity, etc. and there are newer methods such as whole-genome sequencing, etc.  . | Done (End of discussion) |
| **Reviewer #3:** |  |
| **ABSTRACT;** Line 1-2: “The occurrence of multidrug resistant (MDR) bacterial pathogens may become a significant worry, particularly during the last coronavirus disease-19 (COVID-19) pandemic.” Comment: This sentence needs to be re-constructed. The pandemic has not ended. It has only slowed. Here is a suggestion: "Since the peak of the coronavirus disease-19 (COVID-19) pandemic, concerns around multidrug resistant (MDR) bacterial pathogens have increased" | Done |
| Line 4-6: “This bacterial pathogen might acquire specific virulence plasmids that contain aminoglycoside-resistant genes, which may result in the dissemination of serious infections”  Comment: dissemination is not the most appropriate word in this sentence | corrected |
| Line 14-16: “Therefore, there is a need for continuous implementation of effective control measures to monitor the occurrence of MDR pathogens in such situations.” Comment: In which situation? Since it is abstract, it is helpful to be explicit. | Done |
| INTRODUCTION Paragraph 1- Line 7: “During the last coronavirus disease 2019 (COVID-19) pandemic..” Comment: Again, the pandemic is far from over. Although infections are now far between | Done |
| Paragraph 1- Line 13-15: “Evidences from Europe countries and the United States have reported on the link between the COVID-19..” Comment: European countries and not Europe countries | Done |
| Paragraph 2- Line 13: “Other also mechanisms of resistance to aminoglycosides found in Gram-negative bacteria are uptake reduction or decreasing cell permeability besides methylating 16S RNA in ribosomes.” Comment: Delete “Other” from the beginning of this sentence | Corrected |
| Paragraph 2- Line 21-24: “The present study was carried out to identify aimed to identify the frequency of aminoglycoside resistance genes in clinical isolates of MDR K. pneumoniae collected from tertiary hospitals during the COVID19 pandemic” Comment: Delete “identify aimed to” | Corrected |
| Comment: Break the large paragraphs in the introduction to make it easier to read. | done |
| MATERIALS AND METHODS Bacterial Identification: Comment: There was no justification for why the samples were collected between April 2020 and January 2021. Why the choice of hospital too? This was not stated. Why did the research stop at collecting 220 pathogenic Gram-negative bacilli? Why not more or less? The result section provided adequate visuals to convey the insights from the data collection and analysis procedure. | explained |
| Comment: there are two conclusion sections and that is not helpful. One conclusion is enough for the article. | done |
| “second” CONCLUSION section Paragraph 1: Line 1: “The study concluded that MDR K. pneumoniae collected during COVID-19 pandemic in Makkah hospitals was highly resistant to aminoglycosides and other commonly used antibiotics.” Comment: “..during COVID_19…” should be rewritten to reflect that the period of data collection was at the peak of the pandemic and COVID-19 is far from over. The way it is phrased here indicates a past occurrence. | The whole conclusion was edited |
| General comments The objective of the study was clearly stated The method section was underwhelming. The authors did not provide enough information about their process? | Updated |
| The researchers mentioned that the project had no ethical implication, but the pathogenic Gram-negative bacilli were collected from human fluids and their gender split was recorded. How did this scale through the institutional review board of their affiliated institutions of higher learning? | We deleted the gender split records from the results, as they have no significant roles in the study. |
| The first paragraph of the result section should be included in the materials and methods section. | done partially |
| The conclusion section was hurried. The manuscript should be proofread and rearranged for cohesive flow. | done |
