# Supplementary material for: Characterization of aminoglycoside resistance genes in multidrug-resistant Klebsiella pneumoniae collected from tertiary hospitals during the COVID-19 pandemic
Source: PLoS One. 2023 Jul 28;18(7):e0289359. doi: 10.1371/journal.pone.0289359 (PMC10381092; doi:10.1371/journal.pone.0289359)
Supplement: Attachment — Submitted filename: rebuttal lette.docx [file pone.0289359.s002.docx]

**Responses to Comments:**

| **Journal requirements:** | **Response:** |
| --- | --- |
| Please review your reference list to ensure that it is complete and correct. | Done |
| If you have cited papers that have been retracted, please include the rationale for doing so in the manuscript text, or remove these references and replace them with relevant current references. | The withdrawn reference was removed and replaced with relevant reference. |
| Any changes to the reference list should be mentioned in the rebuttal letter that accompanies your revised manuscript. | The reference 37 was replaced by another one. |
| If you need to cite a retracted article, indicate the article’s retracted status in the References list and also include a citation and full reference for the retraction notice. | Citation for the withdrawn (retracted)was article deleted |
| **Reviewers' comments:** | |
| **Reviewer #2:** |  |
| Thank you for addressing the previous comments. The article is now clearer, however, some revisions still need to be made. | OK |
| Lines 182-184: this statement is not clear and the two references refer to a pre-COVID era. The authors should clarify the statement. | clarified |
| Lines 219-221: The flow would be better if this section was moved somewhere after Conclusions. | Right, but we prefer to be here as it shows limitations rather than conclusion. |
| Lines 221-227: Although the authors attempted to address an earlier comment, there is room for further improvement in this section. The current presentation of the limitations of PCR and the advantages of whole genome sequencing seems to undervalue the use of PCR in the analysis, potentially devaluing the entire study. It would be beneficial to provide a more balanced discussion that acknowledges the limitations of PCR but also justifies its usage in the context of this study in conjunction with highlighting the advantages of whole genome sequencing for future studies. | Done |
| Line 221: the authors should correct the typo in "methjod" | Done |
